# Propensity for Bistability of Bursting and Silence in the Leech Heart Interneuron

**DOI:** 10.3389/fncom.2018.00005

**Published:** 2018-02-06

**Authors:** Tatiana Dashevskiy, Gennady Cymbalyuk

**Affiliations:** ^1^Neuroscience Institute, Georgia State University, Atlanta, GA, United States; ^2^Center for Integrative Brain Research, Seattle Children's Research Institute, Seattle, WA, United States

**Keywords:** multistability, coexistence, bifurcation diagrams, neuromodulation, bursting activity, Homoclinic bifurcation, Andronov Hopf bifurcation

## Abstract

The coexistence of neuronal activity regimes has been reported under normal and pathological conditions. Such multistability could enhance the flexibility of the nervous system and has many implications for motor control, memory, and decision making. Multistability is commonly promoted by neuromodulation targeting specific membrane ionic currents. Here, we investigated how modulation of different ionic currents could affect the neuronal propensity for bistability. We considered a leech heart interneuron model. It exhibits bistability of bursting and silence in a narrow range of the leak current parameters, conductance (*g*_*leak*_) and reversal potential (*E*_*leak*_). We assessed the propensity for bistability of the model by using bifurcation diagrams. On the diagram (*g*_*leak*_, *E*_*leak*_), we mapped bursting and silent regimes. For the canonical value of *E*_*leak*_ we determined the range of *g*_*leak*_ which supported the bistability. We use this range as an index of propensity for bistability. We investigated how this index was affected by alterations of ionic currents. We systematically changed their conductances, one at a time, and built corresponding bifurcation diagrams in parameter planes of the maximal conductance of a given current and the leak conductance. We found that conductance of only one current substantially affected the index of propensity; the increase of the maximal conductance of the hyperpolarization-activated cationic current increased the propensity index. The second conductance with the strongest effect was the conductance of the low-threshold fast Ca^2+^ current; its reduction increased the propensity index although the effect was about two times smaller in magnitude. Analyzing the model with both changes applied simultaneously, we found that the diagram (*g*_*leak*_, *E*_*leak*_) showed a progressively expanded area of bistability of bursting and silence.

## Introduction

Both single neurons and neuronal networks ubiquitously exhibit multistability of activity regimes. As a result of this coexistence of regimes, a neuron or a neuronal network could be switched from one regime to another by noise or by a short transient perturbation such as a pulse of synaptic current (Guttman et al., 1980; Paydarfar et al., 2006). Multistability has been implicated in an assortment of brain functions. In information processing and maintaining short-term memory, multistable neurons can play roles of the memory units and the toggle switches (Marder et al., 1996; Egorov et al., 2002; Williams et al., 2002; Loewenstein et al., 2005; Durstewitz and Seamans, 2006; Tahvildari et al., 2007). In motor control, the multistability of interneurons may pertain to the robust and flexible operation of multifunctional central pattern generators (CPGs), the oscillatory neuronal networks controlling different rhythmic motor patterns, like crawling and swimming in the medicinal leech (Getting, 1989; Marder, 1994; Jing and Weiss, 2001; Sutton et al., 2004; Venugopal et al., 2007; Briggman and Kristan, 2008; Schwabedal et al., 2014; Lyttle et al., 2017). Coexistence of silent and tonic spiking regimes exhibited by spinal motoneurons plays an important role in posture maintenance (Hounsgaard et al., 1984; Conway et al., 1988; Eken and Kiehn, 1989; Hounsgaard and Kiehn, 1989; Kiehn and Eken, 1998; Lee and Heckman, 1998b; Carlin et al., 2000; Perrier and Hounsgaard, 2000). Besides its functional roles, multistability of neuronal networks can accompany pathological conditions such as epilepsy (Foss and Milton, 2000; Hahn and Durand, 2001; Fuentealba et al., 2005; Fröhlich and Bazhenov, 2006; Takeshita et al., 2007; Krishnan et al., 2015). In most cases, the mechanisms underlying multistability and the factors leading to multistability are not well understood.

Invertebrate nervous systems provide the prime advantage of gaining insights into the dynamics of multistable systems on the cellular level since their neurons can be uniquely identified by position, morphology, and electrical activity. The phenomenon of bistability in neuronal dynamics was predicted theoretically and confirmed experimentally in various identified invertebrate neurons. Classic examples are the theoretical studies that revealed the bistability of tonic spiking and silence in the squid giant axon (Rinzel, 1978a; Best, 1979; Guttman et al., 1980; Paydarfar et al., 2006) and multistability of tonic spiking and multiple bursting regimes in the R15 neuron in Aplysia (Canavier et al., 1994; Lechner et al., 1996; Butera, 1998; Newman and Butera, 2010). Here, we focused on bistability of bursting and silence in a well-studied, leech generic neuron, an oscillator leech heart interneuron (HN) (Stent et al., 1979; Calabrese et al., 1995, 2016; Hill et al., 2001; Cymbalyuk et al., 2002; Kueh et al., 2016). In our previous work, we predicted that the leech heart interneuron could exhibit bistability of bursting and silence under conditions with elevated leak conductance, although the range of parameters exhibiting bistability is very narrow (Cymbalyuk et al., 2002; Malashchenko et al., 2011a; Marin et al., 2013). We hypothesize that to reveal this bistability experimentally we need to identify neuromodulatory conditions expanding this range.

Multistability of neurons is usually reported as a result of neuromodulation of membrane currents. For example, in motoneurons bistability results from cellular membrane dynamics, and synaptic input plays the role of a toggle-switch between two regimes (Kiehn and Eken, 1998). Such cellular bistability is an endogenous property of motoneurons that can be recruited when necessary by neuromodulators targeting the dynamics of intrinsic ionic currents (Hounsgaard and Kiehn, 1985; Conway et al., 1988; Lee and Heckman, 1998a, 1999; Shapiro and Lee, 2007). Numerous studies concentrate on assessing the partaking of different ionic currents in the dynamics producing multistability (Hounsgaard and Kiehn, 1989; Hsiao et al., 1997; Lee and Heckman, 1998a; Hughes et al., 1999; Williams et al., 2002). The open question which we address here is: Can different currents be evaluated with respect to the propensity of a single neuron for multistability?

The bifurcation theory provides powerful tools for studies of multistability on the cellular level. It allows one to determine specific biophysical parameters and the range of their variation which would support coexistence of particular regimes of activity. For example, the coexistence of tonic spiking and silence in the dynamics of a single neuron has been extensively studied in models using bifurcation analysis (Rinzel, 1978a,b; Best, 1979; Lee and Heckman, 1998a, 1999; Hahn and Durand, 2001; Williams et al., 2002; Guckenheimer et al., 2005; Tabak et al., 2006; Fernandez et al., 2007; Gutkin et al., 2009; Terman and Ermentrout, 2010; Dovzhenok and Kuznetsov, 2012; Yu et al., 2012; Krishnan et al., 2015). Each observable regime of neuronal activity can be described as an attractor and can be mapped in parameter space by using bifurcation diagrams. These diagrams allow one to determine the borders of parameters where attractors are observed and associate bifurcations of stationary and oscillatory regimes with these borders. The mechanisms underlying multistability specify the unstable regimes that form barriers between the coexisting attractors. Analysis of the bifurcations at which the unstable regimes appear and disappear on the diagrams is a key part of the description of the mechanisms supporting multistability. For example, in cardiac pacemakers and the squid giant axon the threshold separating silent and spiking regimes is the stable manifold of a saddle orbit, which represents unstable subthreshold oscillations (Rinzel, 1978a; Landau et al., 1990). The saddle orbit appears at the subcritical Andronov-Hopf bifurcation and disappears at the saddle-node bifurcation for periodic orbits when injected current is varied. These excitable cells, which were initially exhibiting a single observable regime, could turn bistable after the modulation of membrane ionic currents. For instance, the bifurcation analysis of the Hodgkin-Huxley model predicted that bistability could occur if a steady depolarizing current is applied or if the external K^+^ concentration is elevated (Rinzel, 1978a; Hahn and Durand, 2001). In a cardiac cell, application of the depolarizing current also leads to bistability (Jalife and Antzelevitch, 1980).

In this article, we investigate the bistability of bursting and silent regimes of a leech heart interneuron. This bistability was reported in our previous studies of a neuronal model (Cymbalyuk et al., 2002; Malashchenko et al., 2011a; Marin et al., 2013). These studies lead us to the problem of identifying biologically relevant conditions under which this bistability can be revealed experimentally. We considered the well-studied model of the leech heart interneuron. It contains eight voltage-gated currents and a leak current (Hill et al., 2001). All currents were measured except for the fast sodium current. Their dynamics were described based on Hodgkin-Huxley formalism and assembled into a canonical model (Hill et al., 2001). To investigate the model behavior, the reversal potential and conductance of the leak current were used as the controlling parameters. Depending on their values, the model exhibited tonic spiking, bursting activity, subthreshold oscillations, or hyperpolarized and depolarized silent regimes. For the narrow range of the leak current parameters, the model exhibited bistability of bursting activity and silence (Cymbalyuk et al., 2002; Malashchenko et al., 2011a). We suggest that the width of this range can be used as an index evaluating a neuronal propensity for bistability. We assume that the larger the area of bistability bounded by the leak current parameters, the more probable it is to find these neurons bistable experimentally.

Since bistability was observed within a small range of the leak conductance, we looked for the conditions that could expand this range. In our previous work we determined the mechanism supporting the coexistence of bursting and silence. Similar to the mechanism of bistability of tonic spiking and silence detected in cardiac cells and the squid giant axon, it was based on the saddle periodic orbit. By varying a controlling parameter, the leak conductance, we found that the saddle orbit originates at a sub-critical Andronov-Hopf bifurcation. The increase of the parameter led to the termination of the saddle orbit through a homoclinic bifurcation. These two bifurcations bounded the range of the leak current parameters where the saddle orbit existed (Malashchenko et al., 2011a) and thus limited the range of coexistence of bursting and silence.

In the present work, we investigated the leech heart interneuron model to ascertain the conditions that increase the propensity of these neurons for bistability. We applied a general method that allows one to identify how up- and down- regulations of currents affect neuronal bistability (Barnett et al., 2013). We used the knowledge about the mechanism supporting the bistability and investigated the effects of modulation of every voltage-gated ionic conductance on the propensity of a neuron for bistability. The results of this article were included into the dissertation of Tatiana Dashevskiy (Malaschenko) (Malaschenko, 2011).

## Materials and methods

**Figure 1:**
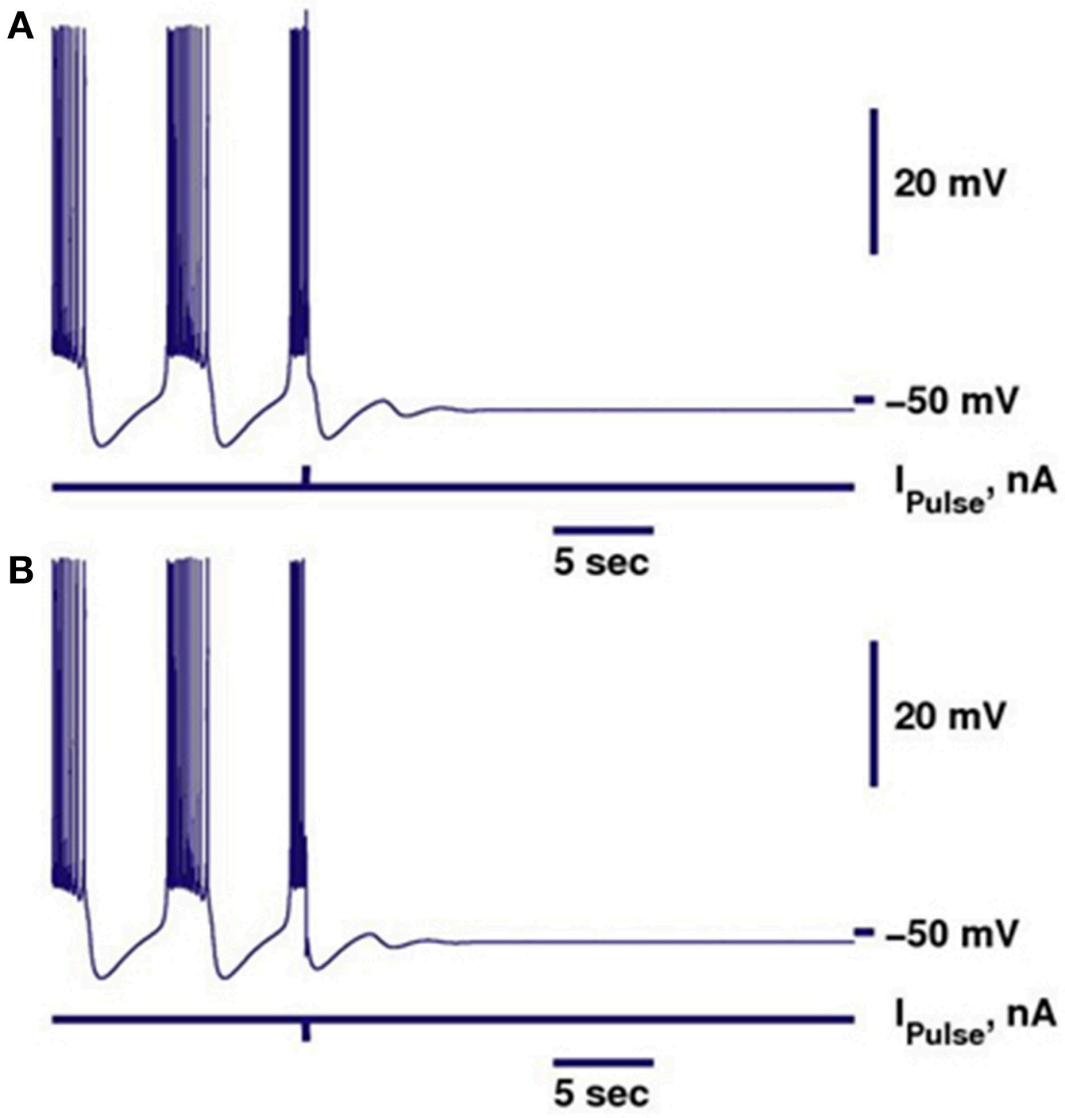
Perturbation of the bursting activity of a bistable neuron by a pulse of current can switch the regime into the silent regime. Bursting is shown in dark blue. Switch from bursting into silence can be performed by either depolarizing **(A)** or hyperpolarizing **(B)** pulse. Parameters of the model were kept the same as in Cymbalyuk et al. (2002), except for *g*_*leak*_ = 10.7 nS.

**Figure 2:**
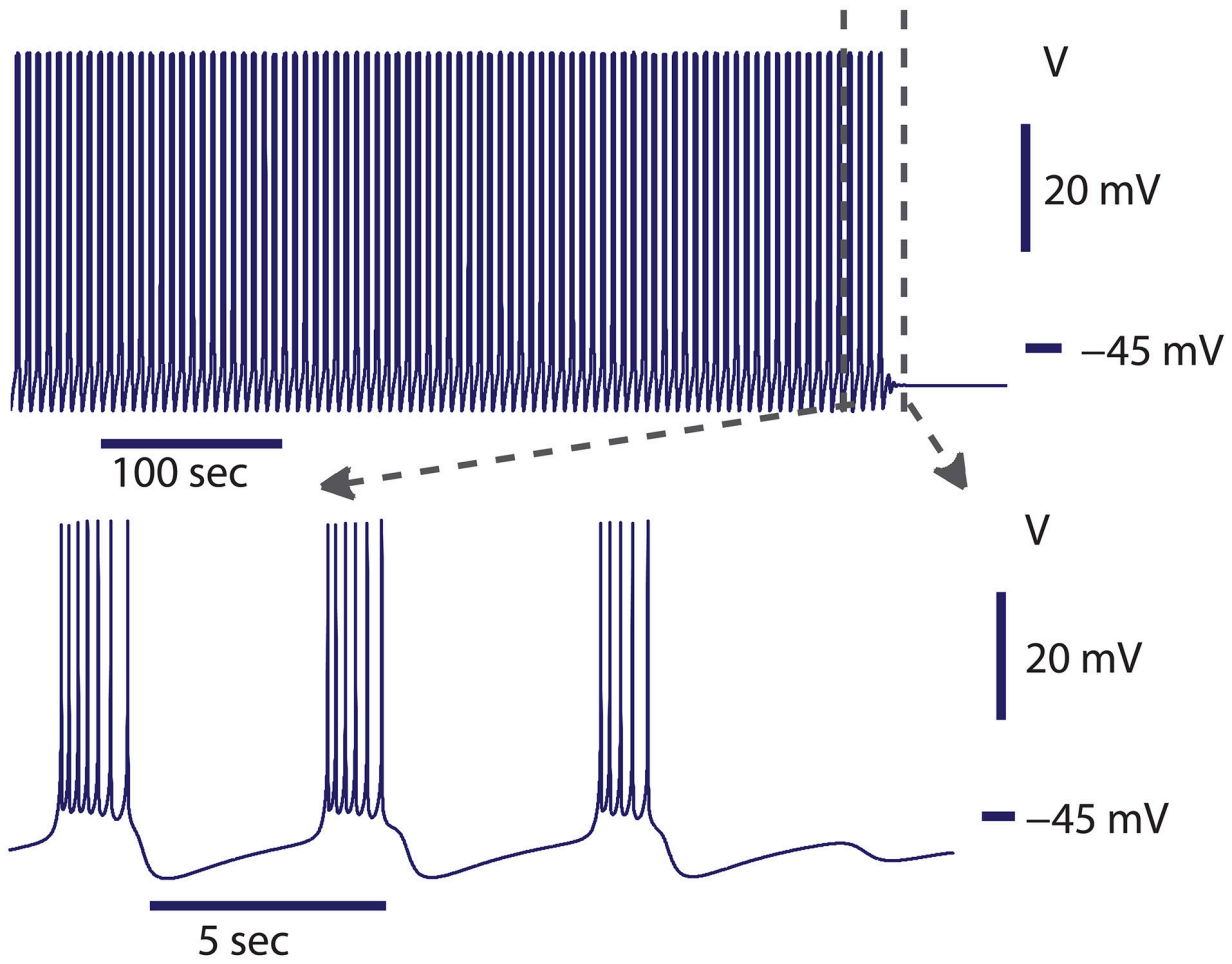
Spontaneous transition from bursting to silence at the transition border from bursting to silence. The model exhibited bursting activity for about 500 s and then the activity spontaneously ceased. The leak current parameters were *g*_*leak*_ = 10.8437 nS, E_leak_ = −0.0635 V. At this value of *g*_*leak*_, the model was located in parameter space near the homoclinic bifurcation for the saddle periodic orbit. The homoclinic bifurcation appeared at *g*_*leak*_ = 10.87 nS. Initial conditions: V = −0.05485488 V, m_CaF_ = 0.007453999, h_CaF_ = 0.3851188, m_CaS_ = 0.0386471, h_CaS_ = 0.3093507, m_K1_ = 0.007837126, h_K1_ = 0.9157689, m_K2_ = 0.05334662, m_KA_ = 0.1961155, h_KA_ = 0.209315, m_h_ = 0.3366125, m_P_ = 0.1307736, m_Na_ = 0.02026809, h_Na_ = 0.999996, and parameters g_leak_ = 10.8437 nS.

The biophysical model of the leech heart interneuron is a single compartment model based on Hodgkin-Huxley formalism (Hill et al., 2001). It contains eight voltage-gated conductances describing two types of sodium currents, fast I_Na_ and persistent I_P_, two low-threshold calcium currents, slow inactivating I_CaS_ and fast I_CaF_, three potassium currents that are delayed rectifier I_K1_, non-inactivating I_K2_ and transient I_KA_, and hyperpolarization-activated cationic current I_h_. The dynamics of currents through the membrane is described by a system of 14 differential equations. Parameters of the models had been chosen so that the activity produced by the model is close to the activity recorded experimentally. The bistability of bursting and silence in this model could be induced by up-regulation of the leak conductance. In this paper, it was elevated to 10.7 nS. To obtain trajectories in Figures 1, 2, the integration of models was performed by a variable-step, variable-order methods based on the numerical differentiation formulae by using a Matlab ODES solver ode15s, Mathworks Inc. (Shampine and Reichelt, 1997). Absolute and relative tolerances were 10^−8^ and 10^−9^, correspondingly. Initial and maximal step sizes were 0.01 and 1, correspondingly. To demonstrate the bistability of bursting and silence in a single neuron, a negative square pulse of current was applied while neuron was initially in a bursting regime. Pulse was delivered near the first spike of a burst. The duration of the pulse was set to 0.03 s.

**Figure 3:**
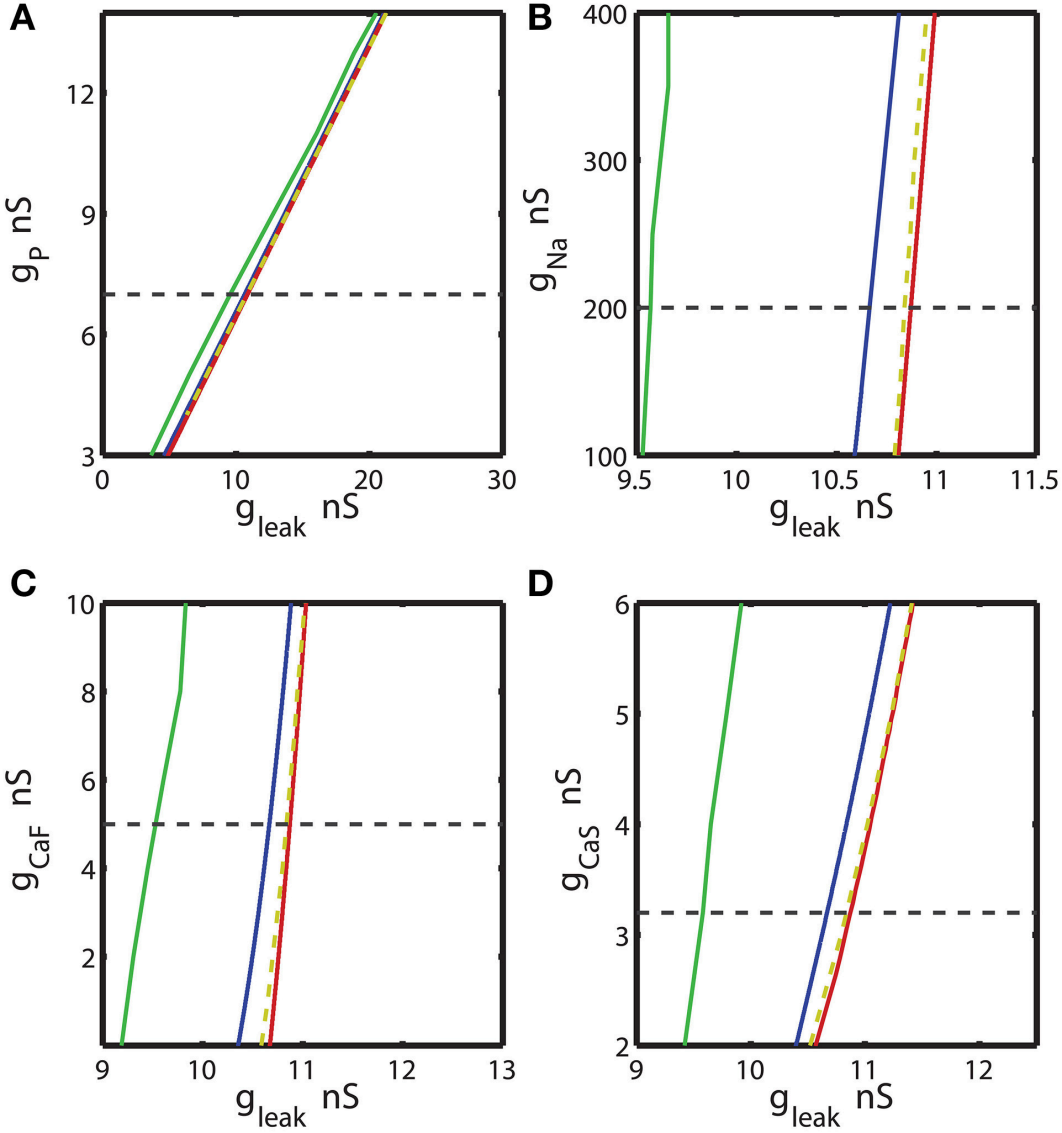
The two parameter bifurcation diagrams mapping the bursting and silent regimes with the variations of the maximal conductances of I_P_, I_Na_, I_CaF_, I_CaS_ vs. g_leak_. Up- and down-regulation of the conductances of inward currents change the index of propensity which is defined as the width of the range of g_leak_ supporting bistability observed for fixed value of the current conductance *ḡ*_*i*_. For each value of *ḡ*_*i*_ index of propensity was calculated. The blue curve marks the sub-critical Andronov Hopf bifurcation, the red curve represents a homoclinic bifurcation. The saddle periodic orbit exists between these two bifurcations. The yellow dashed curve located near homoclinic curve depicts the value of parameters where transition from bursting to silence occurs. The green curve marks the transition from tonic spiking to bursting. The horizontal dashed black line plotted across the diagram corresponds to the canonical parameter of the voltage-gated current conductance under analysis. **(A,B,D)** 2D diagrams are plotted in parameters space of (*ḡ*_*P*_, g_leak_), (*ḡ*_*Na*_, g_leak_), and (*ḡ*_*CaS*_, g_leak_). A slim effect on index of propensity was detected. **(C)** 2D diagram in parameter space of (*ḡ*_*CaF*_, g_leak_) shows that the decrease of *ḡ*_*CaF*_ increases the index of propensity. E_leak_ is fixed to −0.0635 V.

**Figure 4:**
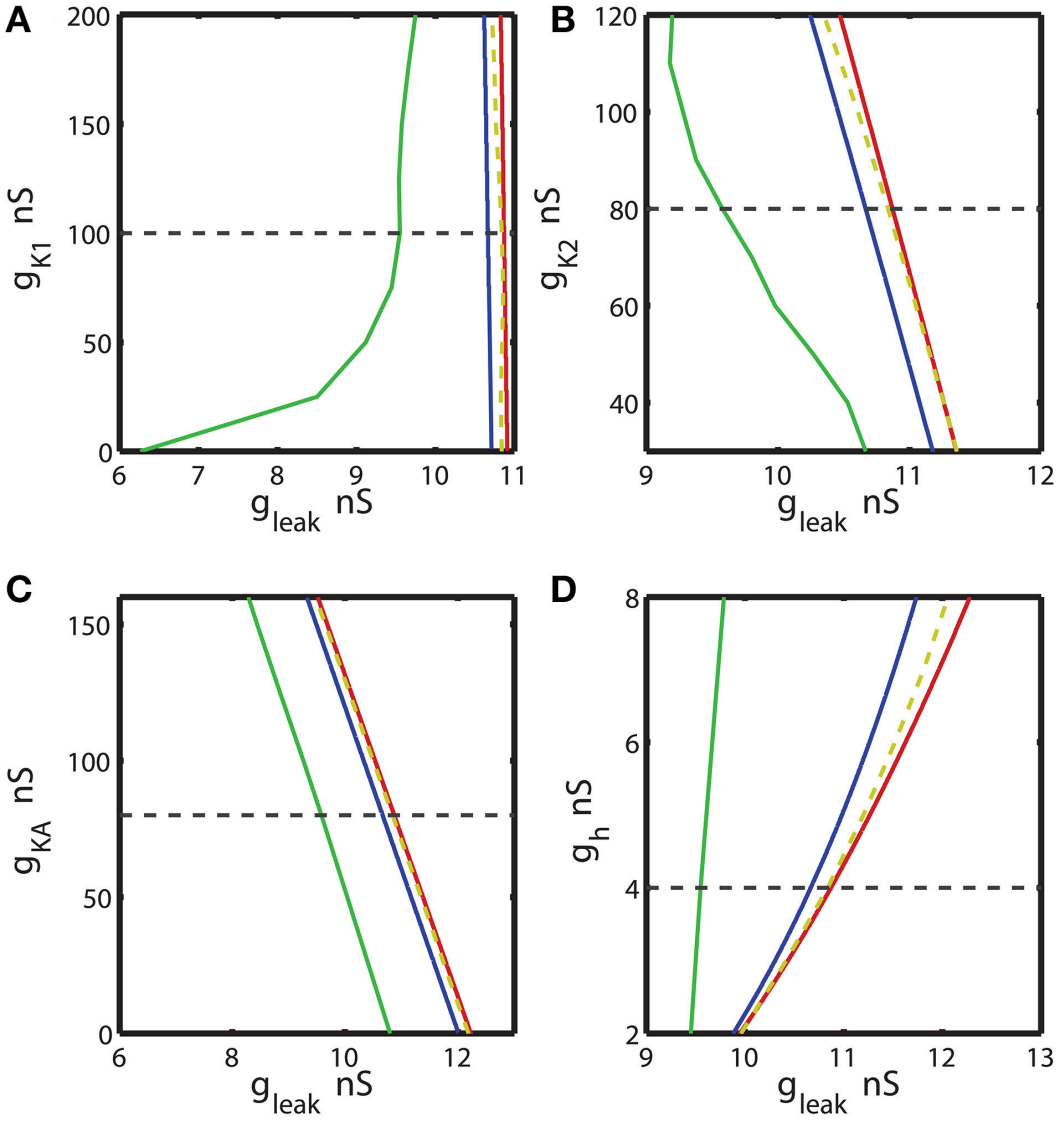
The two parameter bifurcation diagrams mapping the bursting and silent regimes with the variations of the maximal conductances of I_K1_, I_K2_, I_KA_, I_h_ vs. g_leak_. The color and style of the curves are coded as in the Figure 3. **(A–C)** 2D diagrams are plotted in parameters space of (*ḡ*_*K*1_, g_leak_), (*ḡ*_*K*2_, g_leak_), and (*ḡ*_*KA*_, g_leak_) and no substantial change of the index of propensity is detected. **(D)** 2D diagram in parameter space of (*ḡ*_*h*_, g_leak_) shows that the increase of *ḡ*_*h*_ increases the index of propensity. E_leak_ is fixed to −0.0635 V.

A previous study Malashchenko et al. (2011a) showed that the range of E_leak_ and g_leak_ where bistability can be observed is limited by the sub-critical Andronov-Hopf and homoclinic bifurcations. These are co-dimension 1 bifurcations. Within this range the unstable periodic orbit exists. Bifurcation diagrams in Figures 3, 4, **7** were computed using CONTENT software which is freely available at http://www.staff.science.uu.nl/~kouzn101/CONTENT/. We integrated the system by using the Runge-Kutta method of the 4-th order. The tolerance of integration was fixed to 10^−9^ and the minimal step size of integration was set 10^−14^. The software calculates the Andronov-Hopf bifurcation curves and curves of limit cycles with a given period. In Figures 3, 4 the diagrams were calculated for the pairs of parameters: (*g*_*leak*_, *ḡ*_*P*_), (*g*_*leak*_, *ḡ*_*Na*_), (*g*_*leak*_, *ḡ*_*CaF*_), (*g*_*leak*_, *ḡ*_*CaS*_), (*g*_*leak*_, *ḡ*_*K*1_), (*g*_*leak*_, *ḡ*_*K*2_), (*g*_*leak*_, *ḡ*_*KA*_), and (*g*_*leak*_, *ḡ*_*h*_). The maximal value of voltage-gated conductance in the diagrams was constrained by the doubled value of the canonical value of the parameter (Hill et al., 2001). If bursting activity disappears for a smaller value than the doubled value of the canonical value, than the smaller value defines the maximal. The minimal value of a conductance in the diagram was 0 nS, unless the bursting activity disappears for a larger value of the conductance. The homoclinic bifurcation curves in Figures 3, 4 were computed using the procedure which includes 1D and 2D bifurcation analysis. First, we calculated a curve of the equilibria. The main bifurcation parameter was *g*_*leak*_. The maximum step size of the change of *g*_*leak*_ was set to 0.001 nS. We detected the sub-critical Andronov-Hopf bifurcation point where the stable stationary state, representing the silent regime, lost stability. At this point, an unstable sub-threshold periodic orbit collapsed onto the stationary state. Starting from the sub-critical Andronov-Hopf point, we traced the evolution of the unstable periodic orbit. For the orbit continuation, the number of traced points on a periodic orbit was set to 120. The amplitude in the CONTENT window “switch data” was 10^−5^. As we systematically increased *g*_*leak*_, the period of the orbit grew logarithmically toward infinity. We continued this procedure until the orbit had period of 100 s. The obtained value of *g*_*leak*_ approximates a homoclinic bifurcation value (Malashchenko et al., 2011a). We used two parameter diagrams to calculate the equi-periodic curve where the model exhibited the unstable periodic orbit with the same period of 100 s. One bifurcation parameter was *g*_*leak*_ and the other parameter was the maximum conductance of a voltage-gated current under investigation. To calculate the curve of this constant period we used the tolerance of 10^−9^. We denote this curve as an approximation of the homoclinic bifurcation curve.

The index of the propensity for bistability was defined as the width of the range of *g*_*leak*_ supporting the bistability of bursting and silence. The index of propensity is bounded by two critical values of *g*_*leak*_, one determining the subcritical Andronov-Hopf bifurcation and the other corresponding to the transition from bursting to silence. The last is calculated empirically by integrating the system. We varied each conductance one at the time and detected the index of propensity. The slope of the index of propensity vs. *g*_*leak*_ was used to analyze the effect of each current on the propensity for bistability.

### Model of leech heart interneuron

A leech heart interneuron is modeled as a single isopotential compartment with the membrane conductances represented in terms of the Hodgkin and Huxley formalism (Hill et al., 2001). The dynamics of membrane potential (V) of a neuron are governed by

$$\begin{array}{l} C\frac{dV}{dt}=-(I_{Na}+I_{P}+I_{CaF}+I_{CaS}+I_{h}+I_{K1}+I_{K2}+I_{KA}\\ \;+\;I_{leak}-I_{inj}(t)), \end{array}$$

where C is the total membrane capacitance (0.5 nF), *I*_*ion*_ is an intrinsic voltage-gated current, *I*_*inj*_ is the injected current and *I*_*leak*_ is the leak current. The voltage-gated currents are given by

$$\begin{array}{l} I_{Na}={\bar{g}}_{Na}m_{Na}^{3}h_{Na}(V-E_{Na});I_{P}={\bar{g}}_{P}m_{P}(V-E_{Na});I_{CaF}={\bar{g}}_{CaF}m_{CaF}^{2}h_{CaF}(V-E_{Ca});\\ I_{CaS}={\bar{g}}_{CaS}m_{CaS}^{2}h_{CaS}(V-E_{Ca});I_{K1}={\bar{g}}_{K1}m_{K1}^{2}h_{K1}(V-E_{K});I_{K2}={\bar{g}}_{K2}m_{K2}^{2}(V-E_{K});\\ I_{KA}={\bar{g}}_{KA}m_{KA}^{2}h_{KA}(V-E_{K});\;I_{h}={\bar{g}}_{h}m_{h}^{2}(V-E_{h});\;I_{leak}=g_{leak}(V-E_{leak});I_{inj}(t)=I_{pluse}f(t_{pulse}); \end{array}$$

where *ḡ*_*ion*_ is the maximal conductance, *E*_*ion*_ is the reversal potential of a voltage-gated current, *f*(*t*_*pulse*_) *is 1 during the pulse, otherwise* *f*(*t*_*pulse*_) *is 0, m*_*ion*_ and *h*_*ion*_ are the activation and inactivation gating variables, respectively. These variables are governed by the following equations:

$$\begin{array}{l} \;\frac{dm_{K2}}{dt}=\frac{f_{\infty }(-83,\;0.02,\;V)-m_{K2}}{\tau \left(200,\;0.035,\;0.057,\;0.043,\;V\right)}\\ \;\frac{dm_{P}}{dt}=\frac{f_{\infty }(-120,\;0.039,\;V)-m_{P}}{\tau (400,\;0.057,\;0.01,\;0.2,\;V)}\\ \;\frac{dm_{Na}}{dt}=\frac{f_{\infty }(-150,\;0.029,\;V)-m_{Na}}{0.0001}\\ \;\frac{dh_{Na}}{dt}=\frac{f_{\infty }(500,\;0.030,\;V)-h_{Na}}{\tau_{hNa}(V)}\\ \frac{dm_{CaF}}{dt}=\frac{f_{\infty }(-600,\;0.0467,\;V)-m_{CaF}}{\tau_{mCaF}(V)}\\ \;\frac{dh_{CaF}}{dt}=\frac{f_{\infty }(350,\;0.0555,\;V)-h_{CaF}}{\tau (270,\;0.055,\;0.06,\;0.31,\;V)}\\ \frac{dm_{CaS}}{dt}=\frac{f_{\infty }(-420,\;0.0472,\;V)-m_{CaS}}{\tau (-400,\;0.0487,\;0.005,\;0.134,\;V)}\\ \;\frac{dh_{CaS}}{dt}=\frac{f_{\infty }(360,\;0.055,\;V)-h_{CaS}}{\tau (-250,\;0.043,\;0.005,\;0.134,\;V)}\\ \;\frac{dm_{K1}}{dt}=\frac{f_{\infty }(-143,\;0.021,\;V)-m_{K1}}{\tau (150,\;0.016,\;0.001,\;0.011,\;V)}\\ \;\frac{dh_{K1}}{dt}=\frac{f_{\infty }(111,\;0.028,\;V)-h_{K1}}{\tau (-143,\;0.013,\;0.5,\;0.2,\;V)}\\ \frac{dm_{KA}}{dt}=\frac{f_{\infty }(-130,\;0.044,\;V)-m_{KA}}{\tau (200,\;0.03,\;0.005,\;0.011,\;V)}\\ \;\frac{dh_{KA}}{dt}=\frac{f_{\infty }(160,\;0.063,\;V)-h_{KA}}{\tau (-300,\;0.055,\;0.026,\;0.0085,\;V)}\\ \;\frac{dm_{h}}{dt}=\frac{f_{h\;\infty }(V)-m_{h}}{\tau (-100,\;0.073,\;0.7,\;1.7,\;V)} \end{array}$$

where the steady-state activation and inactivation functions are given by the following function:

$$f_{\infty }(a,b,V)=\frac{1}{1+e^{a(V+b)}}$$

except for the steady-state activation of *I*_*h*_ which is given by

$$f_{h\infty }(V)=\frac{1}{1+2e^{180(V+0.047)}+e^{500(V+0.047)}}$$

All time constants are described by the function $\tau \left(a,b,c,d,V\right)=c+\frac{d}{1+e^{a\left(V+b\right)}}$, except for the inactivation time constants for *I*_*Na*_ and *I*_*CaF*_

$$\begin{array}{l} \;\tau_{hNa}(V)=0.004+\frac{0.006}{1+e^{500(V+0.028)}}+\frac{0.01}{\cosh(300(V+0.027))};\\ \tau_{mCaF}(V)=0.011+\frac{0.024}{\cosh(-330(V+0.0467))}. \end{array}$$

The canonical values of the reversal potentials are E_Na_ = −0.045 V, E_CaS_ = 0.135 V, E_K_ = −0.07 V, E_h_ = −0.021 V, and the maximal conductances are *ḡ*_*Na*_ = 200 nS, *ḡ*_*P*_ = 7nS, *ḡ*_*CaF*_ = 5nS, *ḡ*_*CaS*_ = 3.2nS, *ḡ*_*K*1_nS, *ḡ*_*K*2_ = 80nS, *ḡ*_*KA*_ = 80nS, *ḡ*_*h*_ = 4nS, *g*_*leak*_ = 9.9nS.

## Results

### Switch of activity from bursting to silence

In our previous work, we investigated the mechanisms supporting bistability of bursting and silence in the canonical model of the leech heart interneuron (Malashchenko et al., 2011a). By performing bifurcation analysis, we showed that for a specific set of parameters of conductance and reversal potential of the leak current (*g*_*leak*_, *E*_*leak*_) a silent regime coexisted with bursting activity, and the result was mapped on a two parameter diagram of stationary and oscillatory states. This bistability is supported by a saddle periodic orbit. The stable manifold of this orbit separates basins of attractions of silent and bursting regimes and sets a threshold between them. Figure 1 illustrates the switch from the bursting regime into silence. If the perturbation moved the phase point across the threshold into the basin of attraction of a stable rest state, the bursting activity ceased (Figure 1). If during the perturbation, the phase point did not cross the threshold, the neuron resumed bursting activity. Either a hyperpolarizing or depolarizing pulse of current could produce the switch. The switch could happen if the pulse is appropriately adjusted in terms of amplitude and timing. The saddle periodic orbit appeared at the sub-critical Andronov-Hopf bifurcation when the leak conductance, *g*_*leak*_, progressively increased, and it terminated at the homoclinic bifurcation (Malashchenko et al., 2011a). For the set of parameters near the Andronov-Hopf bifurcation the amplitude of the saddle orbit was very small and the switch from bursting into silence by perturbation was hard to achieve. The amplitude of the saddle orbit grew as it approached a homoclinic bifurcation, and for the parameter values near the homoclinic bifurcation the switch into the silent regime could be achieved easily. The transition from bursting to silence is characterized by long-lasting transient bursting activity (Figure 2). If the parameters are set close to critical values of the transition, the model of the leech heart interneuron can exhibit bursting activity for several hundreds of seconds and then suddenly switch from bursting into silence. In Figure 2, the model exhibits bursting activity for 500 s and after 500 s the bursting ceases. The activity can be temporally restored by applying a pulse of injected current.

In order to locate the area of bistability in a two parameter bifurcation diagram, we mapped two borders demarcating the transition from silence to bursting and the transition from bursting to silence (Figures 3, 4, **7**). The transition from silence to bursting was located by the critical parameters of the Andronov-Hopf bifurcation. The border of the transition from bursting to silence was detected by numerical integration of bursting trajectories for different values of *g*_*leak*_ and *E*_*leak*_ parameters. In order to calculate the parameter values corresponding to the transition from bursting to silence, we had to be cautious about possible co-existence of the bursting and silent regimes; since the neuron's activity depends on the choice of initial conditions. The initial conditions define the state of the neuron as the set of the state variables: the membrane potential, and the activation and inactivation gating variables. In (*g*_*leak*_, *E*_*leak*_) bifurcation diagram, each point indicating the transition from bursting to silence was found by numerical integration of the model. First, the initial conditions and *g*_*leak*_ value corresponding to bursting activity were picked. In order to confirm that the bursting was an attractor, we verified that the neuron exhibited bursting for at least 2,000 s. If bursting persisted, we used the set of the state variables at the last phase point of the trajectory of bursting as the initial conditions for the next trial in which we slightly increased *g*_*leak*_ and again performed integration for 2,000 s. The procedure was repeated until the bursting was not observed anymore. The detected critical *g*_*leak*_ was mapped in the 2D diagrams as a transition point from bursting to silence. We assigned *E*_*leak*_ to different values and, by using the described procedure, calculated the corresponding *g*_*leak*_ values of the transition. The collected (*g*_*leak*_, *E*_*leak*_) parameters were plotted as the right-side border of the area of bistability. The border is located near the homoclinic bifurcation curve that limits the range of parameter values of the existence of the unstable regime in (*g*_*leak*_, *E*_*leak*_) parameter plane. For example, for *E*_*leak*_ = −0.0635 V the bistability was recorded for the *g*_*leak*_ values between 10.67 to 10.84 nS. At the value of *g*_*leak*_ = 10.67 nS the neuron underwent the subcritical Andronov-Hopf bifurcation and the transition from silence to the bursting regime occurs. For values of *g*_*leak*_ larger than 10.84 nS the neuron exhibits only the hyperpolarized silence, the value 10.84 nS indicated the transition point from bursting to the silent regime.

### Effects of modulation of ionic conductances on the propensity for bistability

The bifurcation analysis of the bistable system demonstrated the existence of a saddle orbit, and that the range of bistability is limited by values of *g*_*leak*_ corresponding to the Andronov-Hopf and homoclinic bifurcations at which this saddle orbit originates and terminates (Malashchenko et al., 2011a). The transition from bursting to silence was always located between these bifurcations, usually in the vicinity of the homoclinic bifurcation. We traced the Andronov-Hopf and homoclinic bifurcations and mapped them on 2D diagrams. The leak conductance *g*_*leak*_ and a maximal conductance of each ionic current were used as bifurcation parameters. The purpose was to investigate the relative positions of the sub-critical Andronov-Hopf (AH) and homoclinic (Hom) bifurcation curves and the curve (BSi), defining the transition from bursting into silence, while changing *g*_*leak*_ and the maximal conductance of the investigated ionic current, *ḡ*_*i*_, where the subscript *i* identifies the current. This way, we evaluated how up- and down-regulation of a maximal ionic conductance affected the range of the leak conductance where the bursting and silence coexist and where the saddle orbit exists. The difference between the values of *g*_*leak*_ corresponding to the transition from bursting to silence (BSi) and *g*_*leak*_ corresponding to the sub-critical Andronov-Hopf bifurcation (AH) for the same ionic reversal potential value gives the width of the *g*_*leak*_ range where bistability of bursting and silence can be observed. *We suggest that this width of the* *g*_*leak*_ *range can be used as an index evaluating the neuronal propensity for bistability and call it the propensity index H*. To find the whole range of parameters where bursting existed, the curve of the transition from bursting to tonic spiking activity (BTs) was also calculated.

We calculated the 2D bifurcation diagrams using the bifurcation software CONTENT for the pairs of parameters, the leak conductance and the maximal conductance of an ionic current. For the inward currents, the 2D diagrams, (*g*_*leak*_, *ḡ*_*p*_), (*g*_*leak*_, *ḡ*_*Na*_), (*g*_*leak*_, *ḡ*_*CaF*_) and (*g*_*leak*_, *ḡ*_*CaS*_) were calculated, where *ḡ*_*p*_, *ḡ*_*Na*_, *ḡ*_*CaF*_, *ḡ*_*CaS*_ are the maximal conductances of the persistent Na^+^, fast Na^+^, fast Ca^2+^ and slow Ca^2+^ currents (Figures 3A–D). For the bifurcation diagrams we set the upper and lower boundaries of each maximal ionic conductance, *ḡ*_*i*_ so that the upper boundary of *ḡ*_*i*_ did not exceed its doubled canonical value and the lower boundary was set to zero nS. If the bursting regime disappeared at the value smaller (larger) than the established upper (lower) boundary, this value was used as the upper (lower) boundary. Figure 3A shows the sub-critical Andronov-Hopf and homoclinic bifurcation curves in (*g*_*leak*_, *ḡ*_*p*_) parameter space; the saddle orbit exists within the range of parameters determined by these curves. The corresponding *g*_*leak*_ width (horizontal slice) for the given *ḡ*_*p*_ value was very narrow and stayed relatively constant as *ḡ*_*p*_ was up- or down-regulated from its canonical value of 7 nS. This analysis predicts that the propensity index is not noticeably affected by the change of *ḡ*_*p*_. To locate the range of bistability, we calculated the transition from bursting to silence (BSi) that defined the right-side border of the bistable area. The transition BSi curve passes very close to the homoclinic bifurcation curve. The left border of the bistable range is defined by the sub-critical Andronov-Hopf bifurcation curve. The area of bistability in the 2D diagram was approximately located between the Andronov-Hopf and homoclinic curves, and more precisely it was determined by the Andronov-Hopf and BSi curves. The change of *ḡ*_*p*_ affects the bursting area only slightly that is observed in the width of the range of parameters between BTs and BSi curves. The decrease of *g*_*leak*_ beyond the BTs curve showed that the model can exhibit only tonic spiking activity.

Let us now consider the influence of modulation of the fast Na^+^ current on the propensity index. The increase of *ḡ*_*Na*_ from the canonical value, 200 nS, up to 400 nS only slightly decreased the width of *g*_*leak*_ range between the sub-critical Adronov-Hopf and homoclinic bifurcation curves (Figure 3B). Similar to the analysis performed for *ḡ*_*p*_ in Figure 3A we calculated the BTs (transition from bursting to tonic spiking), AH, BSi, and Hom curves. According to the diagram in Figure 3B the bursting area and the area of bistability were little altered when *ḡ*_*Na*_ was changed. The effect of up- or down-regulation of Ca^2+^ currents on the AH and Hom bifurcation is shown in Figures 3C,D. The down-regulation of *ḡ*_*CaF*_ increased the *g*_*leak*_ interval between the two bifurcation curves, whereas the down-regulation of *ḡ*_*CaS*_ produced a smaller but opposite effect. Considering the *g*_*leak*_ interval between AH and BSi curve, the propensity index *H* increased as *ḡ*_*CaF*_ decreased, and in contrast *H* decreased as *ḡ*_*CaS*_ decreased.

Further, we investigated the effect of four other currents, one predominantly inward and three outward. The hyperpolarization-activated current, I_h_, has the reversal potential of −21 mV and mostly acts as an inward current. Figure 4 shows 2D bifurcation diagrams with different sets of bifurcation parameters: (*g*_*leak*_, *ḡ*_*K*1_), (*g*_*leak*_, *g*_*K*2_), (*g*_*leak*_, *ḡ*_*KA*_) and (*g*_*leak*_, *ḡ*_*h*_), where *ḡ*_*K*1_, *ḡ*_*K*2_, *ḡ*_*KA*_ and *ḡ*_*h*_ are the maximal conductances of the delayed rectifying K^+^, fast K^+^, transient K^+^, and hyperpolarization-activated ionic currents, respectively. The increase of *ḡ*_*K*1_ from 0 to 200 nS had almost no effect on the interval of *g*_*leak*_ values between AH and Hom bifurcation curves (Figure 4A), keeping the index of propensity *H* almost constant. On the other hand, the range of (*g*_*leak*_, *ḡ*_*K*1_) parameters supporting the bursting regime in the model increased significantly as *ḡ*_*K*1_ decreased from the canonical value of 100 to 0 nS. This range is located between BTs and BSi curves. Figures 4B,C show that the changes of *ḡ*_*K*2_ and *ḡ*_*KA*_ conductances had no significant effect on the *g*_*leak*_ interval between AH and Hom bifurcation curves. In the same way, we investigated the effects of modulation of *ḡ*_*h*_. The elevation of *ḡ*_*h*_ strongly affected both the area between AH and BSi and the *g*_*leak*_ interval between the AH and Hom bifurcation curves. For instance, the increase of *ḡ*_*h*_ from the canonical value of 4 nS to 8 nS expands the *g*_*leak*_ interval between bifurcations by a factor of 3.2 (Figure 4D). Moreover, the range of the parameters where the bursting is observed, i.e., the area between BTs and BSi curves also significantly increased with the elevation of *ḡ*_*h*_.

**Figure 5:**
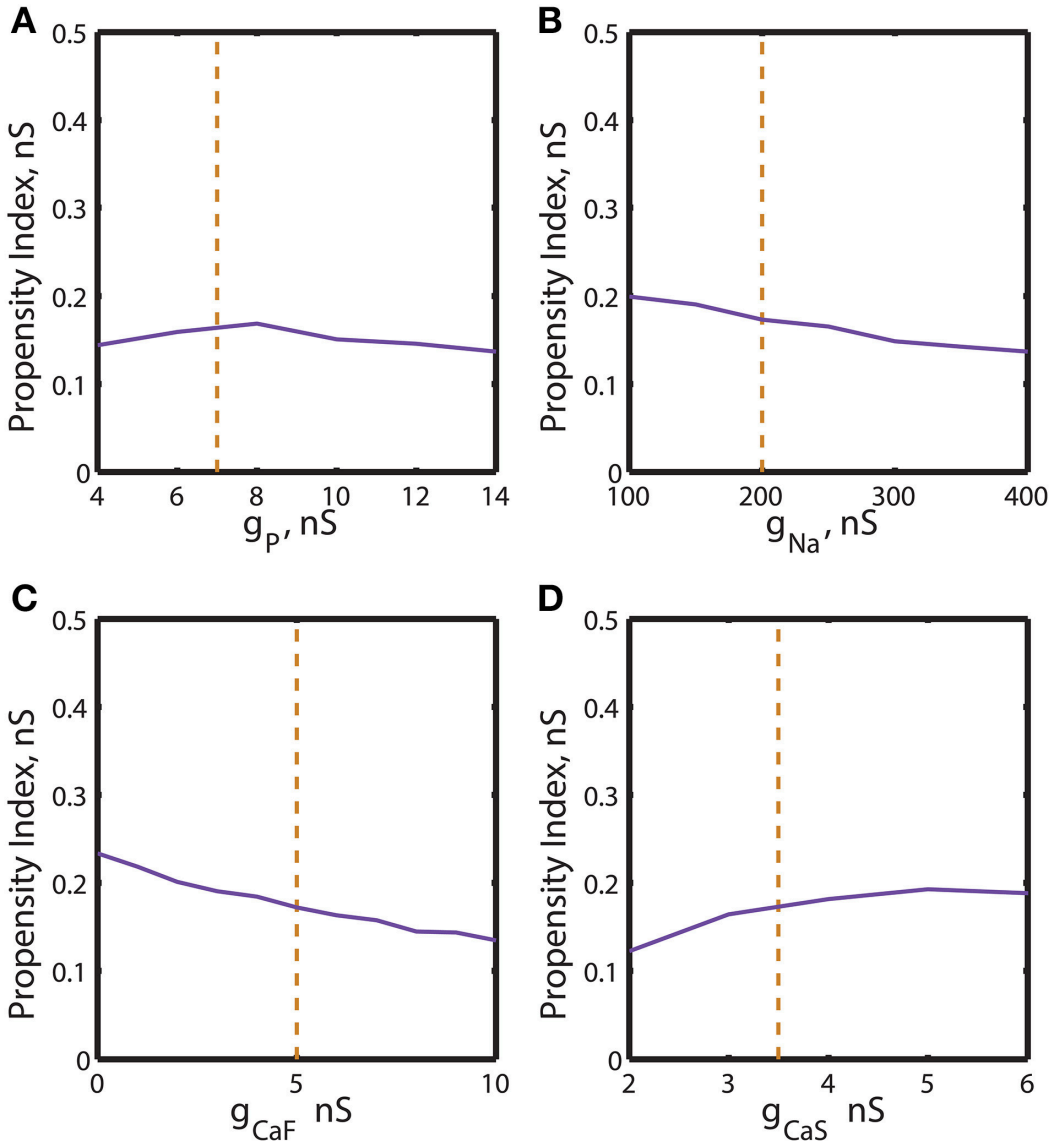
Effect of the variations of the maximal conductances of I_P_, I_Na_, I_CaF_, I_CaS_ on the propensity index for bistability of bursting and silence. The propensity index is defined as the range of g_leak_ where bistability of bursting and silence is observed. The dashed yellow vertical line corresponds to the canonical value of a parameter in the model. **(A)** Manipulation of *ḡ*_*P*_ almost does not affect the index of propensity. **(B)** The decrease of *ḡ*_*Na*_ to 100 nS slightly increases the index of propensity from 0.17 to 0.2. **(C)** The complete reduction of *ḡ*_*CaF*_ increased the index from 0.17 to 0.23. **(D)** The increase of *ḡ*_*CaS*_ almost does not affect index of propensity.

**Figure 6:**
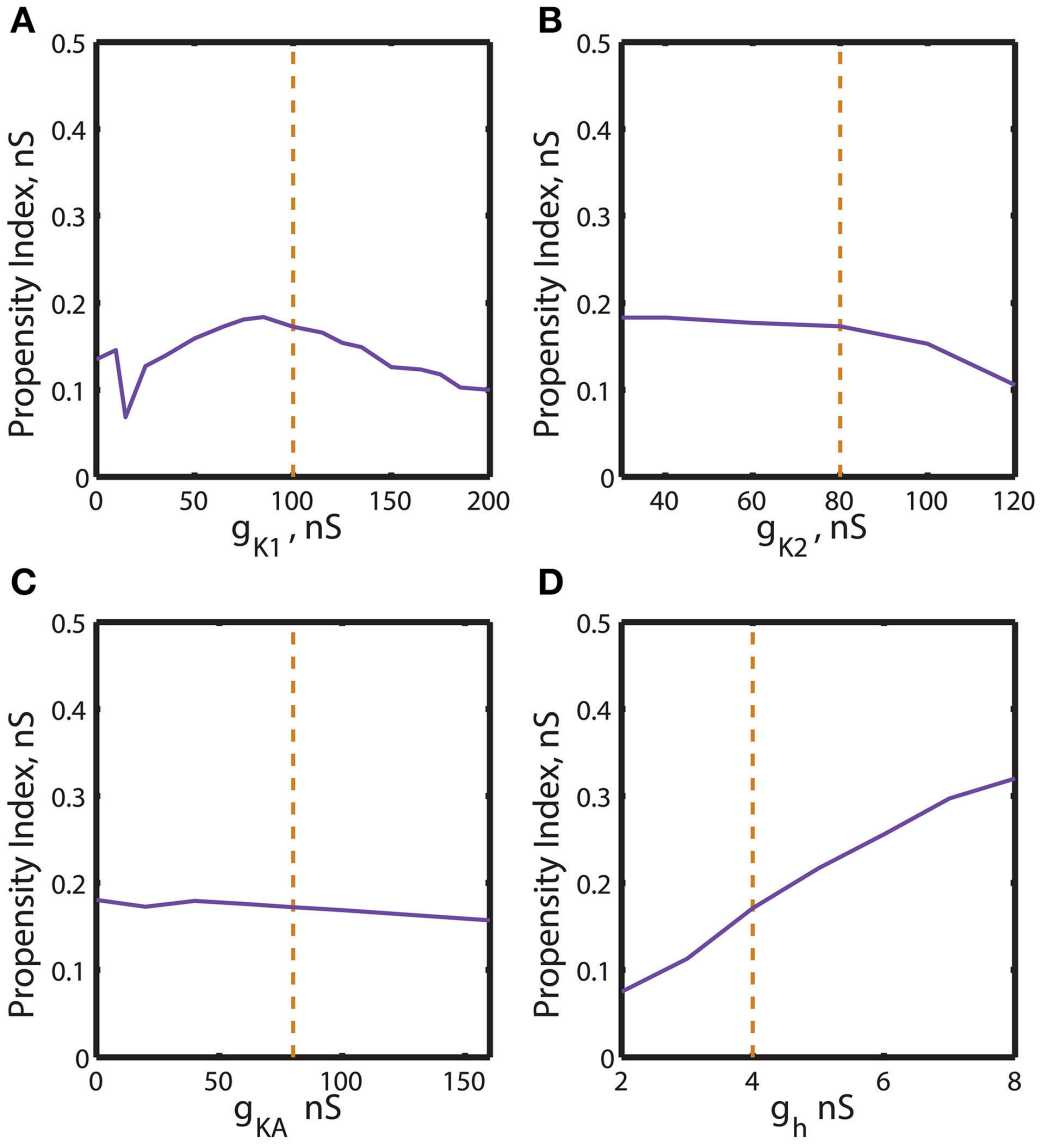
Effect of the variations of the maximal conductances of I_K1_, I_K2_, I_KA_, I_h_ currents on the index of propensity for bistability of bursting and silence. The dashed yellow vertical line corresponds to the canonical value of a parameter in the model. **(A)** Increase or decrease of *ḡ*_*K*1_ mostly decreases the index of propensity. **(B,C)** The changes of *ḡ*_*K*2_ and *ḡ*_*KA*_ do not lead to a significant increase of index of propensity. **(D)** The increase of *ḡ*_*h*_ significantly increases the index of propensity. The change of *ḡ*_*h*_ from 4 to 8 nS increases the index of propensity from 0.17 to 0.31.

Let us further explore the data presented in Figures 3, 4. The bifurcation diagrams in Figures 3, 4 are difficult to assess visually in terms of the change of the propensity index H. Therefore the data from these diagrams were used to construct graphs showing the dependence of the propensity index on the maximal ionic conductances (Figures 5, 6). The value of the propensity index for the canonical set of parameters *H*_*c*_ corresponds to 0.17 nS. A maximal ionic conductance *ḡ*_*i*_ increases the range of bistability of bursting and silence if its up- or down- regulation produced a value of *H* larger than the canonical value *H*_*c*_.

We investigated dependence of the propensity index *H* on *ḡ*_*p*_ (Figure 5A). The value of *H* stays relatively constant near its canonical value and decreases if *ḡ*_*p*_ is further up- or down-regulated. The similar analysis established that the increase of *ḡ*_*Na*_ from 100 to 400 nS decreased the propensity index monotonously (Figure 5B). In contrast, when the dependence of the propensity index on *ḡ*_*CaF*_ and *ḡ*_*CaS*_ was evaluated, we found that the calcium currents affected *H* differently. *H* was increased by up-regulation of *ḡ*_*CaS*_ and reached a maximum near a *ḡ*_*CaS*_ value of 5 nS. In contrast, it was down-regulation of *ḡ*_*CaF*_ that elevated *H* (Figures 5C,D).

A similar procedure was used to summarize the effects of regulation of maximal conductances *ḡ*_*K*1_, *ḡ*_*K*2_, *ḡ*_*KA*_ and *ḡ*_*h*_ on the propensity index. The index varied in a jagged manner with the increase of *ḡ*_*K*1_ from 0 to 200 nS (Figure 6A) and had a maximum near the canonical value of *ḡ*_*K*1_. The increase of *ḡ*_*K*2_ slightly decreased the propensity index, whereas the increase of *ḡ*_*KA*_ almost did not affect it (Figures 6B,C). Interestingly, the increase of *ḡ*_*h*_ from 2 to 8 nS significantly elevates the propensity index (Figure 6D). Notice, among all maximal conductances the increase of *ḡ*_*h*_ affects the most the relative position between AH and Hom bifurcation curves. The data presented in Figures 5, 6 are summarized in Table 1. It contains the maximal and minimal values of the propensity index (H_Max_ and H_Min_) for the regulation of a given maximal ionic conductance and the difference between these values (Δ_H_), Δ_H_ = H_Max_ – H_Min_.

**Table 1:** Minimal and maximal values of propensity index for the different voltage-gated conductances.

	***ḡ_Na_*, nS (100–400)**	***ḡ_P_*, nS (4–14)**	***ḡ_K1_*, nS (0–200)**	***ḡ_K2_*, nS (30–140)**	***ḡ_KA_*, nS (0–160)**	***ḡ_CaF_*, nS (0-10)**	***ḡ_CaS_*, nS (2–6.5)**	***ḡ_h_*, nS (2–8)**
H_min_	0.1366	0.1366	0.0997	0.1054	0.1571	0.1347	0.1223	0.0751
*ḡ*(H_min_)	400	14	200	120	160	10	2	2
H_max_	0.1993	0.17	0.1834	0.1832	0.1803	0.2337	0.1929	0.3200
*ḡ*(H_max_)	100	7	85	40	0	0	5	8
Δ_H_	**0.0627**	0.0334	**0.0837**	**0.0778**	0.0232	**0.099**	**0.0706**	0.2449

*Minimal and maximal values are marked in the table by H_min_ and H_max_. Corresponding conductances to H_min_ and H_max_ are reported as *ḡ*(H_min_) and *ḡ*(H_max_). Values of indexes and conductances are taken from diagrams in Figures 5, 6. The symbol Δ_H_ corresponds to difference between minimal and maximal values of the indexes Δ_H_ = H_max_ − H_min_. Based on the magnitude of Δ_H_ the changes of the propensity index caused by the changes of ionic conductances were classified as small, (marked by gray font), intermediate (marked by black bold font) and significant (underlined black font). Index of propensity for the canonical parameters corresponds to 0.17 nS*.

Summing up, we sorted all currents into three groups based on the achievable gain of the propensity index, Δ_H_: small, intermediate and significant. Comparing the data presented in Table 1, we conclude that the increase of I_p_ and I_KA_ has the smallest effect on the index of propensity. Manipulation of maximal conductances of currents I_CaS_, I_CaF_, I_K1_, I_K2_, I_Na_ produces an intermediate effect on the propensity index. The up-regulation of I_h_ leads to the most significant increase of the propensity index for bistability of bursting and silence. This still leaves the question of how the ionic conductance manipulation will be reflected on (*g*_*leak*_, *E*_*leak*_) diagram. Here, we assume that the larger the area of bistability in the diagram, the easier it would be to find such neurons bistable.

### Changes in the 2d bifurcation diagram of stationary and oscillatory states when I_h_ and I_CaF_ are modified

The critical values of parameters (*g*_*leak*_, *E*_*leak*_) defining the Andronov-Hopf and homoclinic bifurcation curves limit the range of parameters (*g*_*leak*_, *E*_*leak*_) where the bistability of bursting and silence can be observed (Malashchenko et al., 2011a). The area of bistability defined in (*g*_*leak*_, *E*_*leak*_) bifurcation diagram for the canonical set of ionic current parameters was used as a control. We compare the ranges of bistability under different ionic current modifications. We assume that if up- or down-regulation of ionic currents modifies the area of bistability such that it becomes larger than the control, then the more feasible it will be to record the bistability in an experimental setting.

**Figure 7:**
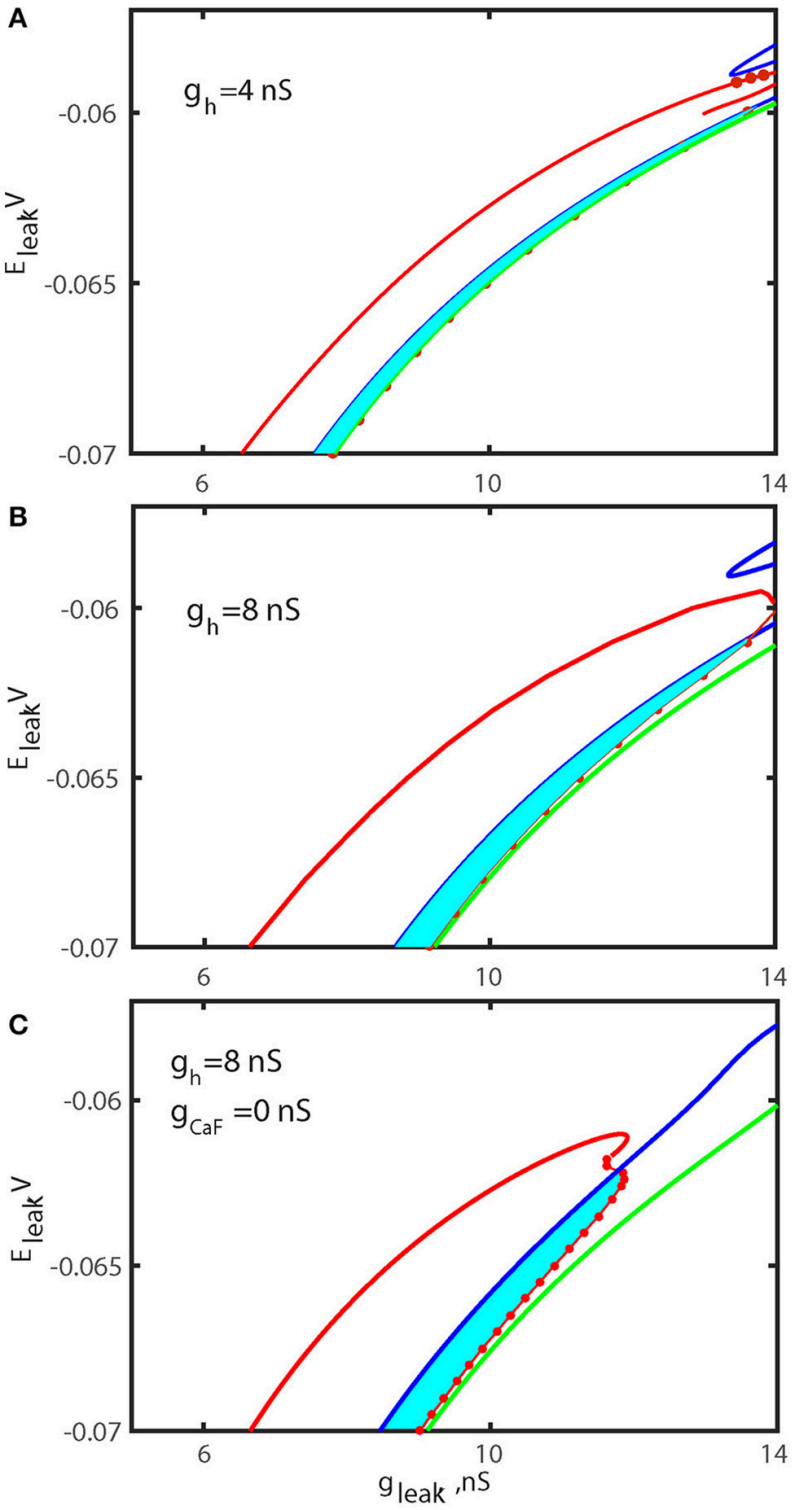
Bifurcation diagrams of the oscillatory and rest regimes in parameter space (g_leak_, E_leak_) for different *ḡ*_*h*_ and *ḡ*_*CaF*_. The sub-critical Andronov-Hopf bifurcation of the hyperpolarized rest state (silent regime) is shown by the blue curve and marks the boundary where silent regime loses stability. The green curve corresponds to the homoclinic bifurcation of the unstable subthreshold oscillations at which the oscillations disappear. The red dots are numerically found points where the transition from bursting activity into silence occurs. The area between the blue curve and the section of the red curve with the dots marks the parameter regime where bursting coexists with rest state. The left section of the red curve locates period-doubling bifurcation of the large amplitude periodic spiking and identifies the transition from tonic spiking to bursting. The diagrams are computed for *ḡ*_*h*_ = 4 nS **(A)**, *ḡ*_*h*_ = 8 nS **(B)**, and *ḡ*_*h*_ = 8 nS and *ḡ*_*CaF*_ = 0 nS **(C)**. The increase of *ḡ*_*h*_ increases index of propensity and the area of bistability. The width of bi-stable area is increased by factor 1.8 for canonical value of E_leak_ = −0.0635V. In **(C)** *ḡ*_*h*_ and *ḡ*_*caF*_ are changed to simultaneously increase the index of propensity. The elimination of fast Ca^2+^ current and the increase of I_h_ current remarkably expanded the width of bistable area.

To test our hypothesis that the up-regulation of *ḡ*_*h*_ significantly increases the range of the bistability, we computed the bifurcation diagrams using the leak current parameters for the value of *ḡ*_*h*_ fixed to 8 nS (Figure 7B) and compared it with the control diagram (Figure 7A). The diagrams depict two areas of parameters values, one supporting the bursting activity and the other supporting the bistability of bursting and silence. These diagrams also depict the sub-critical AH and Hom bifurcation curves. The diagram in Figure 7B indicates that the elevation of *ḡ*_*h*_ significantly increases the area of bistability of bursting and silence in the (*g*_*leak*_, *E*_*leak*_) diagram.

The data presented in Table 1 show that modulations of five currents can be classified as having an intermediately strong effect on the propensity index: I_CaS_, I_CaF_, I_K1_, I_K2_, I_Na_. Among them, the strongest effect is observed with the down regulation of *ḡ*_*CaF*_. Therefore, we investigated whether the superposition of two modulatory conditions could increase the range of bistability even more. We calculated the (*g*_*leak*_, *E*_*leak*_) bifurcation diagram with *ḡ*_*h*_ up-regulated to 8 nS, *ḡ*_*CaF*_ set to 0 nS. Figure 7 shows that the range of bistability is increased in the Figure 7C compared to the Figures 7A,B.

## Discussion

Multistability of oscillatory and silent regimes is a common phenomenon exhibited by excitable systems such as neurons and cardiac pacemakers (Jalife and Antzelevitch, 1980; Hounsgaard et al., 1984; Conway et al., 1988; Eken and Kiehn, 1989; Hounsgaard and Kiehn, 1989; Landau et al., 1990; Hsiao et al., 1997; Kiehn and Eken, 1998; Lee and Heckman, 1998a, 1999; Carlin et al., 2000; Perrier and Hounsgaard, 2000). Studying neuronal dynamics on the cellular level allows thorough investigation of the mechanisms supporting bistability, defined as coexistence of two observable regimes, i.e., attractors, in the phase state space. It has been the focus of numerous experimental and theoretical studies (Rinzel, 1978a; Guttman et al., 1980; Canavier et al., 1994; Lechner et al., 1996; Hsiao et al., 1997; Butera, 1998; Lee and Heckman, 1998a, 1999; Crunelli et al., 2005; Cymbalyuk and Shilnikov, 2005; Cymbalyuk et al., 2005; Shilnikov et al., 2005; Fröhlich and Bazhenov, 2006; Newman and Butera, 2010; Malashchenko et al., 2011a,b; Dovzhenok and Kuznetsov, 2012; Barnett et al., 2013; Marin et al., 2013; Krishnan et al., 2015). A classic example of a bistable system is the squid giant axon exhibiting co-existence of tonic spiking and silence under conditions of low Ca^2+^ bath concentration (Guttman et al., 1980). Spinal motoneurons from the cat, turtle and frog show bistability of tonic spiking and silent regimes (Hounsgaard and Kiehn, 1989; Hsiao et al., 1997). Bistability of tonic spiking and silence was observed in neurons from layer V of the Entorhinal cortex and in the Purkinje neurons in rats (Egorov et al., 2002; Williams et al., 2002; Loewenstein et al., 2005; Tahvildari et al., 2007).

Theoretical analysis of biophysically realistic models can accurately predict whether particular neurons are capable of producing coexisting regimes of activity; and bifurcation analysis allows one to investigate the dynamical mechanisms supporting multistability. Because bistability requires an unstable regime, the mechanisms supporting it can be classified according to the types of separating regimes involved. Typically, a separating regime is either a saddle equilibrium or a saddle orbit. For example, the stable manifold of the saddle equilibrium separates tonic spiking and silence observed in the simplified model of the cerebellar Purkinje neurons (Loewenstein et al., 2005; Fernandez et al., 2007). On the other hand, the stable manifold of the saddle periodic orbit is also a common cause of bistability (Rinzel, 1978a,b; Hahn and Durand, 2001; Shilnikov et al., 2005; Fröhlich and Bazhenov, 2006; Malashchenko et al., 2011a,b; Dovzhenok and Kuznetsov, 2012). Up until recently, most studies have been focused on the bistability of spiking and silence. We are interested in mechanisms of the bistability of bursting and silence that recently has been investigated in a leech heart interneuron model (Malashchenko et al., 2011a,b; Marin et al., 2013).

Here, we show that by using the knowledge of the mechanism, we can elucidate the propensity of the neuron for bistability and the effects of modulations of different ionic currents on it. Since bistability requires presence of an unstable regime, one could asses the range of bistability by determining the bounds of a biophysical controlling parameter where the unstable regime exists. The knowledge of the range of parameters supporting bistability allows us to introduce a measure of the propensity for bistability. We define the propensity index as the width of the range of the controlling parameter where bistability can be observed. As the controlling parameter, one can choose among many biophysical parameters, e.g. the conductance or reversal potential of any ionic current of interest. Because of its special role in the dynamics of excitable cells, following Barnett et al. (2013) in this work, we used the conductance of the leak current as the controlling parameter defining the propensity index. We suggest that the larger the range of values of the leak conductance supporting bistability, the more probable it is to find neurons in the bistable state.

The leak conductance was singled out as such due to the following rationale. The leak current plays an important physiological role in controlling the excitability of neurons. In contrast to other conductances, the leak conductance does not depend on the membrane potential, yet it is a target of modulation. Biophysically, the leak current is carried through multiple channels. Channels with mixed ionic permeability to Na^+^, Cl^−^, K^+^, and Ca^2+^ ions, channels permeable to K^+^ such as TASK channels or channels with primarily Na^+^ permeability such as NALCN are examples of leak channels that control neuronal resting membrane potential (Bayliss et al., 2003; Lu et al., 2007; Koizumi et al., 2010). Depending on the composition of these channels, neuromodulators can reduce or augment the leak conductance. For example, the TASK leak channels are pH-sensitive, and an increase of the extracellular pH up-regulates the leak conductance (Washburn et al., 2002; Larkman and Perkins, 2005). Neurotransmitters including serotonin and noradrenalin close TASK channels, leading to the decrease of the total leak conductance (Sirois et al., 2002; Perrier et al., 2003).

In biophysical models, the activity of leech heart interneurons is highly sensitive to modifications of the leak current (Cymbalyuk et al., 2002; Marin et al., 2013). The notion of such sensitivity is supported by the experiments showing that the activity of leech heart interneurons depends on the method of recording. A leech heart interneuron pharmacologically isolated with bicuculline produces tonic spiking activity if the recording is made intracellularly, whereas it exhibits bursting activity when recording is performed extracellularly (Cymbalyuk et al., 2002). It has been suggested that the intracellular recording with a sharp microelectrode introduces an additional shunting component to the leak current. Similarly, sensitivity to the method of recording has also been reported in *Xenopus* neurons (Aiken et al., 2003). The intracellular recording with a sharp microelectrode shows that *Xenopus* neurons fire single short burst or single spikes in response to a depolarizing pulse of current, whereas the whole-cell recording shows tonic spiking activity in response to the same stimulation.

The universality and special role of the leak current parameters has been exploited in analysis of neuronal dynamics in a variety of models (Guckenheimer et al., 2005). Typically low dimensional systems that are based on Hodgkin-Huxley formalism can be dissected in different time scales, slow and fast (Rinzel, 1985; Bertram et al., 1995; Guckenheimer et al., 2005). Such slow-fast decomposition allows consideration of the slow variable as constant within the fast time scale and using it in turn as a controlling parameter. In neuronal models containing slow currents the leak current could be used as a tool to classify the dynamic regime of a model. Slow currents could be approximately considered as constant on a fast time scale, and the slow currents can be lumped together as a component of the leak current (Guckenheimer et al., 2005). Guckenheimer et al. (2005) applied this idea to describe the neuronal dynamics in several models for which the Na^+^ current is the dominant fast current. The authors considered models of the *Aplysia* neuron, the thalamic relay neuron, a simplified model of the leech heart interneuron, and a neuron from the respiratory center. They detected bifurcations of a fast subsystem that describe the transitions between spiking and silent phases. The analysis was presented in the form of 2D bifurcation diagrams depicting the regions of oscillatory and stationary states.

In this work, we measured the propensity of a neuron for bistability in terms of the leak conductance. The mechanism has been thoroughly studied in our previous works (Malashchenko et al., 2011a,b). Similarly to the Rinzel mechanism (Rinzel, 1978a), the mechanism supporting bistability of bursting and silence requires the presence of a saddle periodic orbit allowing separation of the stable stationary state (silence) and bursting regime (Malashchenko et al., 2011a,b). The range of controlling parameters supporting the bistability is bounded by the two bifurcations at which the saddle orbit appears and disappears. This orbit exists in a range of leak current parameters that is bounded by the sub-critical Andronov-Hopf and homoclinic bifurcations. In this study, we investigated the effect of up- or down-regulation of each current on the bounds limiting the separating regime and on the propensity index. The impact of every current was classified as small, intermediate or significant (Table 1). Only one current, the hyperpolarization-activated current, was shown to significantly increase the propensity index. The second most influential current, the fast Ca^2+^ current, I_CaF_, leads to a moderate increase of the propensity index. Based on our modeling study, we predict that by manipulating these currents experimentally one could reveal the bistability of bursting and silence in a single leech heart interneuron experimentally.

By analyzing how up- or down-regulations of ionic currents affect propensity index, we could assess whether certain neuromodulators promote multistability. In some neurons, it has been shown that neuromodulators could lead to multistability. For example, serotonin induces bistability of spinal motoneurons in different animals (Hounsgaard and Kiehn, 1989; Perrier and Hounsgaard, 2000). Bistability of tonic spiking and silent regimes was found in the presence of serotonin in an *in vitro* preparation of the spinal cord of the turtle. Serotonin promotes the depolarized silent regime and supports Na^+^-dependent tonic spiking activity. By blocking the Na^+^ current with TTX, the depolarized rest state was shown to be maintained by an L-type calcium current and a Ca^2+^-dependent mechanism. A perturbation of the neuron by a brief pulse of current can switch the regime of activity between the hyperpolarized and depolarized rest states (Perrier and Hounsgaard, 2000; Perrier et al., 2000). In the leech heart interneuron, there are two identified Ca^2+^ currents, slow and fast. Our analysis of the model indicated that these currents have different influence on the propensity for bistability: the increase of slow Ca^2+^ conductance weakly increases the propensity for bistability of bursting activity and silence, whereas the increase of fast Ca^2+^ conductance reduces the propensity.

Through the augmentation of ionic currents, neuromodulators play an important role in controlling motor behavior. In the medicinal leech, the endogenous peptide myomodulin has been shown as a modulator of bursting activity of the leech heart interneurons. The myomodulin decreases the period of bursting activity by increasing the conductance of h-current and decreasing the Na^+^/K^+^ pump current (Tobin and Calabrese, 2005). Based on our prediction that h-current leads to a stronger propensity for bistability, we hypothesize that myomodulin will increase the propensity of a heart interneuron for bistability. In other biological systems, the role of the h-current in bistability could be different, e.g., the bistability of tonic spiking and silence of cerebellar Purkinje neurons observed *in vitro*. Some studies show the blockade of this current induces bistability (Williams et al., 2002) whereas another reported bistability of Purkinje neurons in the presence of h-current (Loewenstein et al., 2005). The ionic mechanism of the Purkinje cell bistability has been suggested to rely on non-inactivating inward currents such as the persistent Na^+^ current that supports the depolarized tonic spiking regime (Williams et al., 2002). In contrast, although not directly comparable, our results suggest that persistent Na^+^ current plays an insignificant role in affecting the propensity for bistability of bursting and silence. The apparent discrepancy might be related to the difference in the separating regimes supporting bistability in the Purkinje cell (a saddle rest state) and the leech heart interneuron (a saddle orbit).

Several studies have indicated that multistability may play an important role in organizing multifunctional Central Pattern Generators (CPGs). These CPGs can generate different motor programs such that the switch between different regimes can be achieved either by external stimuli or by neuromodulators (Venugopal et al., 2007; Briggman and Kristan, 2008; Bondy et al., 2015). For example, Briggman and Kristan (2008) suggest that the crawling and swimming CPGs of the leech could be an example of a multifunctional network. The authors show that the vast majority of neurons are involved in both behaviors, swimming or crawling. The modeling of multistable systems can open a new vista onto the understanding of dynamics of currents and control of multistability. The method described in this work can be applied to other neuronal models if the mechanism and the separating regime supporting bistability are known. Concerning our model, we found that only two currents substantially affected the index of the propensity for bistability. These results suggest that under the conditions reported here the coexistence of bursting and silence in an isolated leech heart interneuron could be revealed experimentally.

## Author contributions

TD and GC: provided substantial contribution to conception and design, acquisition of data, analysis and interpretation of data, and writing of the paper. Both authors approved the final version for publication.

### Conflict of interest statement

The authors declare that the research was conducted in the absence of any commercial or financial relationships that could be construed as a potential conflict of interest.

## References

[B1] Aiken S. P. Kuenzi F. M. Nicholas D. (2003). Xenopus embryonic spinal neurons recorded in situ with patch-clamp electrodes - conditional oscillators after all? Eur. J. Neurosci. 18, 333–343. 10.1046/j.1460-9568.2003.02755.x 12887415

[B2] Barnett W. O'Brien G. Cymbalyuk G. (2013). Bistability of silence and seizure-like bursting. J. Neurosci. Methods. 220, 179–189. 10.1016/j.jneumeth.2013.08.021 23999174

[B3] Bayliss D. A. Sirois J. E. Talley E. M. (2003). The TASK family: two-pore domain background K+ channels. Mol. Interv. 3, 205–219. 10.1124/mi.3.4.205 14993448

[B4] Bertram R. Butte M. J. Kiemel T. Sherman A. (1995). Topological and phenomenological classification of bursting oscillations. Bull. Math. Biol. 57, 413–439. 10.1007/BF02460633 7728115

[B5] Best E. N. (1979). Null space in the Hodgkin-Huxley Equations. A critical test. Biophys. J. 27, 87–104. 10.1016/S0006-3495(79)85204-2 262379 PMC1328549

[B6] Bondy B. Klishko A. N. Prilutsky B. I. Cymbalyuk G. (2015). Control of cat walking and paw-shake by a multifunctional central pattern generator, in Neuromechanical Modeling of Posture and Locomotion, eds Prilutsky B. I. Edwards D. H. (New York, NY: Springer), 333–359.

[B7] Briggman K. L. Kristan W. B. (2008). Multifunctional pattern-generating circuits. Annu. Rev. Neurosci. 31, 271–294. 10.1146/annurev.neuro.31.060407.125552 18558856

[B8] Butera R. J. (1998). Multirhythmic bursting. Chaos 8, 274–284. 10.1063/1.166358 12779730

[B9] Calabrese R. L. Nadim F. Olsen O. H. (1995). Heartbeat control in the medicinal leech - a model system for understanding the origin, coordination, and modulation of rhythmic motor patterns. J. Neurobiol. 27, 390–402. 10.1002/neu.480270311 7673897

[B10] Calabrese R. L. Norris B. J. Wenning A. (2016). The neural control of heartbeat in invertebrates. Curr. Opin. Neurobiol. 41, 68–77. 10.1016/j.conb.2016.08.004 27589603 PMC5123911

[B11] Canavier C. C. Baxter D. A. Clark J. W. Byrne J. H. (1994). Multiple modes of activity in a model neuron suggest a novel mechanism for the effects of neuromodulators. J. Neurophysiol. 72, 872–882. 10.1152/jn.1994.72.2.872 7983542

[B12] Carlin K. P. Jones K. E. Jiang Z. Jordan L. M. Brownstone R. M. (2000). Dendritic L-type calcium currents in mouse spinal motoneurons: implications for bistability. Eur. J. Neurosci. 12, 1635–1646. 10.1046/j.1460-9568.2000.00055.x 10792441

[B13] Conway B. A. Hultborn H. Kiehn O. Mintz I. (1988). Plateau potentials in alpha-motoneurones induced by intravenous injection of L-dopa and clonidine in the spinal cat. J. Physiol. 405, 369–384. 10.1113/jphysiol.1988.sp017337 3255795 PMC1190980

[B14] Crunelli V. Toth T. I. Cope D. W. Blethyn K. Hughes S. W. (2005). The 'window' T-type calcium current in brain dynamics of different behavioural states. J. Physiol. 562 (Pt 1), 121–129. 10.1113/jphysiol.2004.076273 15498803 PMC1665496

[B15] Cymbalyuk G. S. Calabrese R. L. Shilnikov A. L. (2005). How a neuron model can demonstrate co-existence of tonic spiking and bursting. Neurocomputing 65, 869–875. 10.1016/j.neucom.2004.10.107

[B16] Cymbalyuk G. S. Gaudry Q. Masino M. A. Calabrese R. L. (2002). Bursting in leech heart interneurons: cell-autonomous and network-based mechanisms. J. Neurosci. 22, 10580–10592. 12486150 10.1523/JNEUROSCI.22-24-10580.2002 PMC6758431

[B17] Cymbalyuk G. Shilnikov A. (2005). Coexistence of tonic spiking oscillations in a leech neuron model. J. Comput. Neurosci. 18, 255–263. 10.1007/s10827-005-0354-7 15830162

[B18] Dovzhenok A. Kuznetsov A. S. (2012). Exploring neuronal bistability at the depolarization block. PLoS ONE 7:e42811. 10.1371/journal.pone.0042811 22900051 PMC3416767

[B19] Durstewitz D. Seamans J. K. (2006). Beyond bistability: biophysics and temporal dynamics of working memory. Neuroscience 139, 119–133. 10.1016/j.neuroscience.2005.06.094 16326020

[B20] Egorov A. V. Hamam B. N. Fransen E. Hasselmo M. E. Alonso A. A. (2002). Graded persistent activity in entorhinal cortex neurons. Nature 420, 173–178. 10.1038/nature01171 12432392

[B21] Eken T. Kiehn O. (1989). Bistable firing properties of soleus motor units in unrestrained rats. Acta Physiol. Scand. 136, 383–394. 10.1111/j.1748-1716.1989.tb08679.x 2750539

[B22] Fernandez F. R. Engbers J. D. Turner R. W. (2007). Firing dynamics of cerebellar purkinje cells. J. Neurophysiol. 98, 278–294. 10.1152/jn.00306.2007 17493923

[B23] Fröhlich F. Bazhenov M. (2006). Coexistence of tonic firing and bursting in cortical neurons. Phys. Rev. Stat. Nonlin. Soft. Matter Phys. 74(3 Pt 1):031922. 10.1103/PhysRevE.74.031922 17025682

[B24] Foss J. Milton J. (2000). Multistability in recurrent neural loops arising from delay. J. Neurophysiol. 84, 975–985. 10.1152/jn.2000.84.2.975 10938321

[B25] Fuentealba P. Timofeev I. Bazhenov M. Sejnowski T. J. Steriade M. (2005). Membrane bistability in thalamic reticular neurons during spindle oscillations. J. Neurophysiol. 93, 294–304. 10.1152/jn.00552.2004 15331618 PMC2915789

[B26] Getting P. A. (1989). Emerging principles governing the operation of neural networks. Annu. Rev. Neurosci. 12, 185–204. 10.1146/annurev.ne.12.030189.001153 2648949

[B27] Guckenheimer J. Tien J. H. Willms A. R. (2005). Bifurcations in the fast dynamics of neurons: implications for bursting, in Bursting: The Genesis of Rhythm in the Nervous System, eds Coombes S. Bresloff P. C. (London; Singapore; Beijing; Shanghai; Hong Kong; Taipei; Chennai: World Scientific Press), 91–124.

[B28] Gutkin B. S. Jost J. Tuckwell H. C. (2009). Inhibition of rhythmic neural spiking by noise: the occurrence of a minimum in activity with increasing noise. Naturwissenschaften 96, 1091–1097. 10.1007/s00114-009-0570-5 19513592 PMC2727367

[B29] Guttman R. Lewis S. Rinzel J. (1980). Control of repetitive firing in squid axon membrane as a model for a neuroneoscillator. J. Physiol. 305, 377–395. 10.1113/jphysiol.1980.sp013370 7441560 PMC1282979

[B30] Hahn P. J. Durand D. M. (2001). Bistability dynamics in simulations of neural activity in high-extracellular-potassium conditions. J. Comput. Neurosci. 11, 5–18. 10.1023/A:1011250329341 11524575

[B31] Hill A. A. Lu J. Masino M. A. Olsen O. H. Calabrese R. L. (2001). A model of a segmental oscillator in the leech heartbeat neuronal network. J. Comput. Neurosci. 10, 281–302. 10.1023/A:1011216131638 11443286

[B32] Hounsgaard J. Hultborn H. Jespersen B. Kiehn O. (1984). Intrinsic membrane properties causing a bistable behaviour of alpha-motoneurones. Exp. Brain Res. 55, 391–394. 6086378 10.1007/BF00237290

[B33] Hounsgaard J. Kiehn O. (1985). Ca++ dependent bistability induced by serotonin in spinal motoneurons. Exp. Brain Res. 57, 422–425. 10.1007/BF00236551 2578974

[B34] Hounsgaard J. Kiehn O. (1989). Serotonin-induced bistability of turtle motoneurones caused by a nifedipine-sensitive calcium plateau potential. J. Physiol. 414, 265–282. 10.1113/jphysiol.1989.sp017687 2607432 PMC1189141

[B35] Hsiao C. F. Trueblood P. R. Levine M. S. Chandler S. H. (1997). Multiple effects of serotonin on membrane properties of trigeminal motoneurons *in vitro*. J. Neurophysiol. 77, 2910–2924. 10.1152/jn.1997.77.6.2910 9212246

[B36] Hughes S. W. Cope D. W. Toth T. I. Williams S. R. Crunelli V. (1999). All thalamocortical neurones possess a T-type Ca^2+^ ‘window’ current that enables the expression of bistability-mediated activities. J. Physiol. 517 (Pt 3), 805–815. 10.1111/j.1469-7793.1999.0805s.x 10358120 PMC2269384

[B37] Jalife J. Antzelevitch C. (1980). Pacemaker annihilation: diagnostic and therapeutic implications. Am. Heart J. 100, 128–130. 10.1016/0002-8703(80)90289-6 7386358

[B38] Jing J. Weiss K. R. (2001). Neural mechanisms of motor program switching in Aplysia. J. Neurosci. 21, 7349–7362. 11549745 10.1523/JNEUROSCI.21-18-07349.2001 PMC6762995

[B39] Kiehn O. Eken T. (1998). Functional role of plateau potentials in vertebrate motor neurons. Curr. Opin. Neurobiol. 8, 746–752. 10.1016/S0959-4388(98)80117-7 9914232

[B40] Koizumi H. Smerin S. E. Yamanishi T. Moorjani B. R. Zhang R. Smith J. C. (2010). TASK channels contribute to the K+-dominated leak current regulating respiratory rhythm generation *in vitro*. J. Neurosci. 30, 4273–4284. 10.1523/JNEUROSCI.4017-09.2010 20335463 PMC2950010

[B41] Krishnan G. P. Filatov G. Shilnikov A. Bazhenov M. (2015). Electrogenic properties of the Na(+)/K(+) ATPase control transitions between normal and pathological brain states. J. Neurophysiol. 113, 3356–3374. 10.1152/jn.00460.2014 25589588 PMC4443608

[B42] Kueh D. Barnett W. H. Cymbalyuk G. S. Calabrese R. L. (2016). Na(+)/K(+) pump interacts with the h-current to control bursting activity in central pattern generator neurons of leeches. Elife 5:e19322. 10.7554/eLife.19322 27588351 PMC5010386

[B43] Landau M. Lorente P. Michaels D. Jalife J. (1990). Bistabilities and annihilation phenomena in electrophysiological cardiac models. Circ. Res. 66, 1658–1672. 10.1161/01.RES.66.6.1658 2344667

[B44] Larkman P. M. Perkins E. M. (2005). A TASK-like pH- and amine-sensitive ‘leak’ K+ conductance regulates neonatal rat facial motoneuron excitability *in vitro*. Eur. J. Neurosci. 21, 679–691. 10.1111/j.1460-9568.2005.03898.x 15733086

[B45] Lechner H. A. Baxter D. A. Clark J. W. Byrne J. H. (1996). Bistability and its regulation by serotonin in the endogenously bursting neuron R15 in Aplysia. J. Neurophysiol. 75, 957–962. 10.1152/jn.1996.75.2.957 8714668

[B46] Lee R. H. Heckman C. J. (1998a). Bistability in spinal motoneurons *in vivo*: systematic variations in persistent inward currents. J. Neurophysiol. 80, 583–593. 10.1152/jn.1998.80.2.583 9705452

[B47] Lee R. H. Heckman C. J. (1998b). Bistability in spinal motoneurons *in vivo*: systematic variations in rhythmic firing patterns. J. Neurophysiol. 80, 572–582. 10.1152/jn.1998.80.2.572 9705451

[B48] Lee R. H. Heckman C. J. (1999). Enhancement of bistability in spinal motoneurons *in vivo* by the noradrenergic alpha1 agonist methoxamine. J. Neurophysiol. 81, 2164–2174. 10.1152/jn.1999.81.5.2164 10322057

[B49] Loewenstein Y. Mahon S. Chadderton P. Kitamura K. Sompolinsky H. Yarom Y.. (2005). Bistability of cerebellar Purkinje cells modulated by sensory stimulation. Nat. Neurosci. 8, 202–211. 10.1038/nn1393 15665875

[B50] Lu B. Su Y. Das S. Liu J. Xia J. Ren D. (2007). The neuronal channel NALCN contributes resting sodium permeability and is required for normal respiratory rhythm. Cell 129, 371–383. 10.1016/j.cell.2007.02.041 17448995

[B51] Lyttle D. N. Gill J. P. Shaw K. M. Thomas P. J. Chiel H. J. (2017). Robustness, flexibility, and sensitivity in a multifunctional motor control model. Biol. Cybern. 111, 25–47. 10.1007/s00422-016-0704-8 28004255 PMC5326633

[B52] Malaschenko T. (2011). Mechanisms of Multistability in Neuronal Models. Ph.D. Dissertation, Physics and Astronomy, Georgia State University.

[B53] Malashchenko T. Shilnikov A. Cymbalyuk G. (2011a). Bistability of bursting and silence regimes in a model of a leech heart interneuron. Phys. Rev. E Stat. Nonlin. Soft. Matter Phys. 84(4 Pt 1):041910. 10.1103/PhysRevE.84.041910 22181178

[B54] Malashchenko T. Shilnikov A. Cymbalyuk G. (2011b). Six types of multistability in a neuronal model based on slow calcium current. PLoS ONE 6:e21782. 10.1371/journal.pone.0021782 21814554 PMC3140973

[B55] Marder E. (1994). Invertebrate neurobiology - polymorphic neural networks. Curr. Biol. 4, 752–754. 10.1016/S0960-9822(00)00169-X 7953569

[B56] Marder E. Abbott L. F. Turrigiano G. G. Liu Z. Golowasch J. (1996). Memory from the dynamics of intrinsic membrane currents. Proc. Natl. Acad. Sci. U.S.A. 93, 13481–13486. 10.1073/pnas.93.24.13481 8942960 PMC33634

[B57] Marin B. Barnett W. H. Doloc-Mihu A. Calabrese R. L. Cymbalyuk G. S. (2013). High prevalence of multistability of rest states and bursting in a database of a model neuron. PLoS Comput. Biol. 9:e1002930. 10.1371/journal.pcbi.1002930 23505348 PMC3591289

[B58] Newman J. P. Butera R. J. (2010). Mechanism, dynamics, and biological existence of multistability in a large class of bursting neurons. Chaos 20:023118. 10.1063/1.3413995 20590314 PMC2902537

[B59] Paydarfar D. Forger D. B. Clay J. R. (2006). Noisy inputs and the induction of on-off switching behavior in a neuronal pacemaker. J. Neurophysiol. 96, 3338–3348. 10.1152/jn.00486.2006 16956993

[B60] Perrier J. F. Alaburda A. Hounsgaard J. (2003). 5-HT1A receptors increase excitability of spinal motoneurons by inhibiting a TASK-1-like K+ current in the adult turtle. J. Physiol. 548(Pt 2), 485–492. 10.1113/jphysiol.2002.037952 12626670 PMC2342869

[B61] Perrier J. F. Hounsgaard J. (2000). Development and regulation of response properties in spinal cord motoneurons. Brain Res. Bull. 53, 529–535. 10.1016/S0361-9230(00)00386-5 11165788

[B62] Perrier J. F. Mejia-Gervacio S. Hounsgaard J. (2000). Facilitation of plateau potentials in turtle motoneurones by a pathway dependent on calcium and calmodulin. J. Physiol. 528(Pt 1), 107–113. 10.1111/j.1469-7793.2000.t01-1-00107.x 11018109 PMC2270105

[B63] Rinzel J. (1978a). On repetitive activity in nerve. Fed. Proc. 37, 2793–2802. 720633

[B64] Rinzel J. (1978b). Repetitive activity and Hopf bifurcation under point-stimulation for a simple FitzHugh-Naguma nerve conduction model. J. Math. Biol. 5, 363–382. 750625 10.1007/BF00276107

[B65] Rinzel J. (1985). Excitation dynamics: insights from simplified membrane models. Fed. Proc. 44, 2944–2946. 2415401

[B66] Schwabedal J. T. Neiman A. B. Shilnikov A. L. (2014). Robust design of polyrhythmic neural circuits. Phys. Rev. E Stat. Nonlin. Soft Matter Phys. 90:022715. 10.1103/PhysRevE.90.022715 25215766

[B67] Shampine L. F. Reichelt M. W. (1997). The MATLAB ODE Suite. SIAM J. Sci. Comput. 18, 1–22. 10.1137/S1064827594276424

[B68] Shapiro N. P. Lee R. H. (2007). Synaptic amplification versus bistability in motoneuron dendritic processing: a top-down modeling approach. J. Neurophysiol. 97, 3948–3960. 10.1152/jn.00084.2007 17409175

[B69] Shilnikov A. Calabrese R. L. Cymbalyuk G. (2005). Mechanism of bistability: tonic spiking and bursting in a neuron model. Phys. Rev. E Statis. Nonlin. Soft Matter Phys. 71 (5 Pt 2):056214. 10.1103/PhysRevE.71.056214 16089641

[B70] Sirois J. E. Lynch C. III. Bayliss D. A. (2002). Convergent and reciprocal modulation of a leak K+ current and I(h) by an inhalational anaesthetic and neurotransmitters in rat brainstem motoneurones. J. Physiol. 541 (Pt 3), 717–729. 10.1113/jphysiol.2002.018119 12068035 PMC2290347

[B71] Stent G. S. Thompson W. J. Calabrese R. L. (1979). Neural control of heartbeat in the leech and in some other invertebrates. Physiol. Rev. 59, 101–136. 10.1152/physrev.1979.59.1.101 220645

[B72] Sutton G. P. Mangan E. V. Neustadter D. M. Beer R. D. Crago P. E. Chiel H. J. (2004). Neural control exploits changing mechanical advantage and context dependence to generate different feeding responses in Aplysia. Biol. Cybern. 91, 333–345. 10.1007/s00422-004-0517-z 15517341

[B73] Tabak J. O'Donovan M. J. Rinzel J. (2006). Differential control of active and silent phases in relaxation models of neuronal rhythms. J. Comput. Neurosci. 21, 307–328. 10.1007/s10827-006-8862-7 16896520

[B74] Tahvildari B. Fransen E. Alonso A. A. Hasselmo M. E. (2007). Switching between On and Off states of persistent activity in lateral entorhinal layer III neurons. Hippocampus 17, 257–263. 10.1002/hipo.20270 17315198

[B75] Takeshita D. Yasuomi S. Sonya B. (2007). Transitions between multistable states as a model of epileptic seizure dynamics. Phys. Rev. E Stat. Nonlin. Soft Matter Phys.75 (5 Pt 1):051925. 10.1103/PhysRevE.75.051925 17677116

[B76] Terman D. H. Ermentrout G. B. (2010). Mathematical Foundations of Neuroscience Interdisciplinary Applied Mathematics. New York, NY: Springer.

[B77] Tobin A. E. Calabrese R. L. (2005). Myomodulin increases Ih and inhibits the NA/K pump to modulate bursting in leech heart interneurons. J. Neurophysiol. 94, 3938–3950. 10.1152/jn.00340.2005 16093342 PMC1560091

[B78] Venugopal S. Travers J. B. Terman D. H. (2007). A computational model for motor pattern switching between taste-induced ingestion and rejection oromotor behaviors. J. Comput. Neurosci. 22, 223–238. 10.1007/s10827-006-0009-3 17072755

[B79] Washburn C. P. Sirois J. E. Talley E. M. Guyenet P. G. Bayliss D. A. (2002). Serotonergic raphe neurons express TASK channel transcripts and a TASK-like pH- and halothane-sensitive K+ conductance. J. Neurosci. 22, 1256–1265. 11850453 10.1523/JNEUROSCI.22-04-01256.2002 PMC6757559

[B80] Williams S. R. Christensen S. R. Stuart G. J. Hausser M. (2002). Membrane potential bistability is controlled by the hyperpolarization-activated current I(H) in rat cerebellar Purkinje neurons *in vitro*. J. Physiol. 539 (Pt 2), 469–483. 10.1113/jphysiol.2001.013136 11882679 PMC2290163

[B81] Yu N. Morris C. E. Joos B. Longtin A. (2012). Spontaneous excitation patterns computed for axons with injury-like impairments of sodium channels and Na/K pumps. PLoS Comput. Biol. 8:e1002664. 10.1371/journal.pcbi.1002664 23028273 PMC3441427

